# Structural Characterization of Non-structural Protein 9 Complexed With Specific Nanobody Pinpoints Two Important Residues Involved in Porcine Reproductive and Respiratory Syndrome Virus Replication

**DOI:** 10.3389/fmicb.2020.581856

**Published:** 2020-11-12

**Authors:** Yan Wang, Rui Li, Songlin Qiao, Jiaxi Wang, Hongliang Liu, Zhijun Li, Hongfang Ma, Lei Yang, Haiyu Ruan, Maoyang Weng, Julian A. Hiscox, James P. Stewart, Yuchen Nan, Gaiping Zhang, En-Min Zhou

**Affiliations:** ^1^Department of Preventive Veterinary Medicine, College of Veterinary Medicine, Northwest A&F University, Yangling, China; ^2^Key Laboratory of Animal Immunology of the Ministry of Agriculture, Henan Provincial Key Laboratory of Animal Immunology, Henan Academy of Agricultural Sciences, Zhengzhou, China; ^3^Department of Infection Biology, Institute of Infection and Global Health, University of Liverpool, Liverpool, United Kingdom

**Keywords:** PRRSV, Nsp9, RdRp, nanobody, SAXS

## Abstract

Porcine reproductive and respiratory syndrome (PRRS), caused by PRRS virus (PRRSV), is a widespread viral disease that has led to huge economic losses for the global swine industry. Non-structural protein 9 (Nsp9) of PRRSV possesses essential RNA-dependent RNA polymerase (RdRp) activity for viral RNA replication. Our previous report showed that Nsp9-specific nanobody, Nb6, was able to inhibit PRRSV replication. In this study, recombinant Nsp9 and Nsp9-Nb6 complex were prepared then characterized using bio-layer interferometry (BLI) and dynamic light scattering (DLS) analyses that demonstrated high-affinity binding of Nb6 to Nsp9 to form a homogeneous complex. Small-angle X-ray scattering (SAXS) characterization analyses revealed that spatial interactions differed between Nsp9 and Nsp9-Nb6 complex molecular envelopes. Enzyme-linked immunosorbent assays (ELISAs) revealed key involvement of Nsp9 residues Ile588, Asp590, and Leu643 and Nb6 residues Tyr62, Trp105, and Pro107 in the Nsp9-Nb6 interaction. After reverse genetics-based techniques were employed to generate recombinant Nsp9 mutant viruses, virus replication efficiencies were assessed in MARC-145 cells. The results revealed impaired viral replication of recombinant viruses bearing I588A and L643A mutations as compared with replication of wild type virus, as evidenced by reduced negative-strand genomic RNA [(−) gRNA] synthesis and attenuated viral infection. Moreover, the isoleucine at position 588 of Nsp9 was conserved across PRRSV genotypes. In conclusion, structural analysis of the Nsp9-Nb6 complex revealed novel amino acid interactions involved in viral RNA replication that will be useful for guiding development of structure-based anti-PRRSV agents.

## Introduction

Porcine reproductive and respiratory syndrome (PRRS), first reported in 1987 in the United States, is one of the most widespread and severe viral disease threats to the global swine industry (Wensvoort et al., 1991; Nan et al., 2017). This disease is characterized by reproductive failures in sows and respiratory diseases in pigs of all ages (Lunney et al., 2010). The etiological agent, PRRS virus (PRRSV), belongs to the genus *Betaarterivirus* of the family *Arteriviridae* within the order *Nidovirales*. PRRSV isolates are divided into two major genotypes: *Betaarterivirus suid 1* (PRRSV-1) and *Betaarterivirus suid 2* (PRRSV-2) (Lin et al., 2020). These two genotypes share less than 70% identity across their entire genomes (Guo et al., 2018). In 2006, an atypical PRRS outbreak caused by a highly pathogenic PRRSV strain (HP-PRRSV) occurred in China, leading to a country-wide epidemic (Tian et al., 2007; Tong et al., 2007).

Porcine reproductive and respiratory syndrome virus is an enveloped RNA virus with a single positive-strand genome of approximately 15 kb. It contains at least ten open reading frames (ORFs), including ORF1a, ORF1ab, ORF2a, ORF2b, ORF3, ORF4, ORF5a, ORF5, ORF6, and ORF7 (Fang and Snijder, 2010; Snijder et al., 2013). ORF1a and ORF1ab occupy three quarters of the genome and encode two large polyproteins. Translation products of ORF1a and ORF1ab, pp1a and pp1ab, respectively, are processed by host and viral proteases into 16 non-structural proteins (Nsps) including Nsp1α/β, Nsp2-6, Nsp7α/β, Nsp8-12, Nsp2TF, and Nsp2N (Fang and Snijder, 2010; Fang et al., 2012). Among these Nsps, Nsp9 is highly conserved (Darwich et al., 2011) and possesses RNA-dependent RNA polymerase (RdRp) activity that is crucial for viral replication and transcription complex (RTC) formation (Snijder et al., 2013), while also contributing to HP-PRRSV virulence (Li et al., 2014). Meanwhile, Nsp9 also interacts with other cellular factors required for PRRSV infection, including annexin A2, retinoblastoma protein and DEAD-box RNA helicase 5 (Dong et al., 2014; Zhao et al., 2015). Thus, the apparent indispensable role played by Nsp9 in PRRSV functions warrants its evaluation as a promising anti-PRRSV target for development of antiviral agents. Indeed, RNA interference-based interventions targeting Nsp9 have already been shown to block PRRSV replication in permissive cell lines (Xie et al., 2014).

Nanobodies (Nbs), also known as camel single-domain antibodies (sdAbs), are derived from the variable region of *Camellidae* heavy chain-only antibodies (VHH) (Hamers-Casterman et al., 1993). Nbs possess various advantages compared to conventional monoclonal antibodies (Hassanzadeh-Ghassabeh et al., 2013), such as small size, ease of genetic manipulation, high specificity and solubility. Moreover, Nbs can be easily produced in prokaryotic or eukaryotic hosts, making them ideal candidates for drug development (Marasco, 2001). Our previous report demonstrated that a PRRSV Nsp9-specific Nb, designated Nb6, inhibited PRRSV replication *in vitro* when expressed intracellularly in PRRSV-permissive cells (Liu et al., 2015). Meanwhile, we have also demonstrated that expressed recombinant fusion proteins incorporating Nb6 fused with cell-penetrating peptides (CPP) exhibited broad antiviral activities against various PRRSV isolates of both genotypes (Wang et al., 2019). However, the antiviral mechanism whereby Nb6 targets Nsp9 to disrupt PRRSV function is unknown. In this study, recombinant PRRSV-Nsp9 and Nsp9-Nb6 complex were prepared then subjected to bio-layer interferometry (BLI) and structure-based biophysical analyses to visualize structural changes associated with Nsp9-Nb6 complex formation. Next, enzyme-linked immunosorbent assays (ELISAs) were conducted to identify key amino acid residues involved in the Nsp9-Nb6 interaction. In addition, we generated recombinant Nsp9-mutant viruses using a reverse genetics-based approach then assessed mutant virus replication efficiencies *in vitro* to determine key residues involved in viral replication.

## Materials and Methods

### Cells and Viruses

Porcine reproductive and respiratory syndrome virus-permissive MARC-145 cells (derived from African green monkey kidney cells) and CRL-2843-CD163 (a cell line stably expressing CD163 in CRL-2843) constructed in our laboratory were maintained in Dulbecco’s modified Eagle’s medium (DMEM; Thermo Fisher Scientific, Waltham, MA, United States) and routinely maintained in Roswell Park Memorial Institute 1640 medium (RPMI 1640, Thermo Fisher Scientific), respectively, supplemented with 10% heat-inactivated fetal bovine serum (FBS; Gibco, Carlsbad, CA, United States) at 37°C in 5% CO_2_. The PRRSV strain used in this study was HP-PRRSV SD16 (GenBank #: JX087437).

### Recombinant Nb6 and Nsp9 Expression and Purification

The sequence of Nb6 was amplified by PCR from template plasmid pEGFP-Nb6 (Liu et al., 2015) using primers listed in Table 1. The resulting fragment was isolated and digested with *Xba*I and *Xho*I (New England Biolabs, Ipswich, MA, United States), then was ligated to pPICZαA expression vector using DNA ligase (New England Biolabs), yielding the construct pPICZαA-Nb6. The construct was transformed into competent *Escherichia coli* DH5α then transformants were screened for growth on low salt LB (LLB) agar plates (0.5% yeast extract, 1% tryptone, 0.5% NaCl, 2% agar) containing 30 μg/ml Zeocin (Thermo Fisher Scientific). Positive colonies were verified by DNA sequencing (Sangon Biotech, Shanghai, China).

**TABLE 1:** Primers used in this study.

Primes	Sequence (5′-3′)^a^	Application
XhoI-U	ATCA*CTCGAG*AAAAGAATGGCCCAGGTGCAGCTGCAGG	pPICZαA-Nb6
XbaI-R	AGCT*TCTAGA*TCAATGATGGTGGTGATGATGTGAGGAGAC	
I588A-F	CCAAAGAAGACAACCGCCACAGATTCACCATCATT	PCR mutagenesis
I588A-R	AATGATGGTGAATCTGTGGCGGTTGTCTTCTTTGG	
D590A-F	AAGACAACCATCACAGCATCACCATCATTCCTA	
D590A-R	TAGGAATGATGGTGATGCTGTGATGGTTGTCTT	
L643A-F	GACAGCTGTGCTTGTGCAGAGTATGATCCTGAAT	
L643A-R	ATTCAGGATCATACTCTGCACAAGCACAGCTGTC	
D646A-F	TTGTTTAGAGTATGCACCTGAATGGTTTGAA	
D646A-R	TTCAAACCATTCAGGTGCATACTCTAAACAA	
Y62A-F	TGGTGGTTCTACAACAGCCGCAGACTCCGTGAA	
Y62A-R	TTCACGGAGTCTGCGGCTGTTGTAGAACCACCA	
D64A-F	TTCTACAACATACGCAGCCTCCGTGAAGGGCCGATT	
D64A-R	AATCGGCCCTTCACGGAGGCTGCGTATGTTGTAGAA	
W105A-F	AGGGCGCGTACAGGCATGGCCTGTACTACGC	
W105A-R	GCGTAGTACAGGCCATGCCTGTACGCGCCCT	
P107A-F	CGCGTACAGTGGTGGGCTGTACTACGCGCGCTGAA	
P107A-R	TTCAGCGCGCGTAGTACAGCCCACCACTGTACGCG	
pBA-F	GCGAACTGACTGCCAAAGAAC	
pBA-R	GCCCGGGGGAAAATGAAACCTC	
ORF7-F	AGATCATCATCGCCCAACAAAAC	RT-qPCR
ORF7-R	GACACAATTGCCGCTCACTA	
β-actin-F	TCCCTGGAGAAGAGCTACGA	
β-actin-R	AGCACTGTGTTGGCGTACAG	
F-6	GTATAGGTGTTGGCTCTATGC	(−) gRNA analysis
R-683	GGGAGCGGCAAGTTGGTTAACAC	
F-12	GTGTTGGCTCTATGCCACGGC	
R-343	TATAAAATAGACCCAGCACCC	
(−) gRNA-actin-F	CTTCCTGGGCATGGAGTCC	β-actin analysis
(−) gRNA-actin-R	GGCGCGATGATCTTGATCTTC	

*^*a*^Mutated nucleotides are underlined. ^*b*^Restriction sites are underlined and shown as italics.*

Competent *Pichia pastoris* strain X-33 cells (Thermo Fisher Scientific) were prepared for DNA transformation according to the manufacturer’s instructions. The plasmid pPICZαA-Nb6 was linearized using *Sac*I (TaKaRa, Dalian, China) then transformed into X-33 via electroporation (1500 V, 25 μF, 200 Ω for 6 ms). After incubation for 1 h at 30°C in 1 M sorbitol without agitation, transformed X-33 cells were cultured on YPD agar plates (1% yeast extract, 2% peptone, 2% glucose, 2% agar) containing 100 μg/ml Zeocin at 30°C for 3 days. Colonies were screened to detect transformants harboring the gene encoding Nb6 integrated within host chromosomal DNA. Positive recombinant X-33 transformants for chromosomally integrated Nb6 were grown in BMGY medium (1% yeast extract, 2% peptone, 100 mM potassium phosphate, pH 6.0, 1.34% YNB, 4 × 10^–5^% biotin, 1% v/v glycerol) to an OD_600_ reading of 2-6 then were cultured in BMMY medium (1% yeast extract, 2% peptone, 100 mM potassium phosphate, pH 6.0, 1.34% YNB, 4 × 10^–5^% biotin, 1% v/v methanol). Protein expression was induced by daily additions of 1% (v/v) methanol for 5 days. After 5 days of induction, the cell culture supernatant was collected and recombinant proteins were purified using a Novagen Ni-NTA column (Merck, Darmstadt, Germany) pre-equilibrated with 20 mM Tris–HCl, pH 8.0, 150 mM NaCl. The target protein was then eluted with 20 mM Tris–HCl, pH 8.0, 150 mM NaCl and 150 mM imidazole and further purified using GE Superdex 75 10/300 GL on GE AKTA^TM^ Pure Chromatography System (GE Healthcare, Pittsburgh, PA, United States) with 20 mM Tris–HCl, pH 8.0 and 150 mM NaCl as elution buffer. Eluted proteins were concentrated using Millipore ultracentrifugation filter tubes (Merck) to 10 mg/ml in 20 mM Tris–HCl, pH 8.0, 150 mM NaCl.

Expression and purification of Nsp9 were performed as reported previously (Liu et al., 2015). Briefly, Nsp9-coding DNA amplified from template plasmid pBAC-SD16FL (Wang C. et al., 2013) was inserted between *NcoI* and *XhoI* sites of pET-28a then the resulting plasmid was transformed into *E. Coli* Transetta strain (Transgen, Beijing, China). Expressed Nsp9 protein was purified using metal affinity chromatography (Ni-NTA column; Novagen) and size-exclusion chromatography (Superdex 200 increase 10/300 GL; GE Healthcare).

### Preparation of Nsp9-Nb6 Complex

Nsp9-Nb6 complex was generated by incubating excess purified Nb6 with purified Nsp9 in buffer containing 20 mM Tris–HCl, pH 8.0, 150 mM NaCl at 4°C overnight. The complex was then isolated via gel filtration chromatography (Superdex 75 10/300 GL; GE healthcare) to remove excess Nb6.

### Bio-Layer Interferometry (BLI) and Dynamic Light Scattering (DLS) Analyses

In order to determine constants for equilibrium dissociation (K_D_), association rate (k_on_) and dissociation rate (k_off_) to understand binding of Nb6 to Nsp9, BLI analyses were carried out using ForteBio’s Octet optical biosensor system (Sartorius, Fremont, CA, United States). Briefly, biotinylated Nsp9 was generated using EZ-Link NHS-LC-LC-Biotin (Thermo Fisher Scientific) then was loaded onto the streptavidin biosensor (Sartorius). Various concentrations of Nb6 (1 μM, 500, 250, 125, and 62.5 nM) were tested.

Dynamic Light Scattering analyses were performed using a DLS measurement instrument (Malvern, Malvern, United Kingdom) equipped with a 2-ml micro-sampling cell maintained at 25°C. The Nsp9-Nb6 complex was diluted to 1.5 μM in buffer containing 20 mM Tris–HCl, pH 8.0, 150 mM NaCl. All samples were filtered through a 0.22-μm Millipore filter membrane to remove any dust particles prior to DLS analyses. Each sample was transferred to a cuvette that was placed into the unit and equilibrated for 2 min at 4°C before measurements were taken.

### Small-Angle X-ray Scattering (SAXS) Assay and Structural Modeling

Small-angle X-ray Scattering measurements of Nsp9 and Nsp9-Nb6 complex molecular envelopes in the buffer containing 20 mM Tris–HCl, pH 8.0, 150 mM NaCl were conducted on the SIBYLS Beamline 12.3.1 at the Shanghai Synchrotron Radiation Facility (SSRF) BL19U2. All data sets were measured with three exposure times (0.5, 1, and 6 s) at 283 K. Three concentrations (2, 3, and 5 mg/ml) of Nsp9 and the complex were used for measurements. Data of buffers were collected between every two protein samples. Scattering data for triplicate solutions were then scaled and average values were subtracted. All curves obtained for the three different solution concentrations were also scaled and merged to generate an average scattering curve. Quality scores of scattering curves were analyzed using the program PRIMUS (Konarev et al., 2003) to assure that no radiation damage and no obvious protein aggregation had occurred before selection of the best sample for use in further analyses. Initial radius of gyration (Rg) values were calculated from Guinier plots and the P(r) distribution function was calculated using the GNOM program (Petoukhov et al., 2012). Low-resolution shapes of Nsp9 and the Nsp9-Nb6 complex in solution were modeled using DAMMIF server (Franke and Svergun, 2009) based on P1 symmetry and an asymmetric unit. Continuous and meaningful shapes were extracted and averaged using DAMAVER server (Volkov and Svergun, 2003). Protein structure models of Nsp9 and Nb6 were predicted using the I-TASSER program^1^ as previously described (Liu et al., 2016) then models were built onto the envelopes using the Chimera program (Pettersen et al., 2004). Figures were generated using PyMOL (Delano, 2002).

### Verification of the Interaction Between Nsp9 and Nb6 *in vitro*

The Nsp9-Nb6 interaction was evaluated by ELISA using 96-well microtiter plates coated with Nsp9 mutant protein (200 ng/well). After incubation with biotinylated Nb6, Nb-bound proteins were detected using horseradish peroxidase (HRP)-conjugated streptavidin (Proteintech, Wuhan, China) with wild type Nsp9 included as the control. Similarly, biotinylated wild type (WT) Nsp9 was utilized to test its binding to the Nb6 mutants.

### Reverse Genetics-Based Mutagenesis of PRRSV

Reverse genetics-based mutagenesis of PRRSV was conducted after DNA from a known infectious viral strain (SD16) was cloned to generate pBAC-SD16 (Wang C. et al., 2013). Amino acid (aa) residues at positions 588, 590, and 643 of Nsp9 were mutated via site-directed mutagenesis using primers listed in Table 1. After cDNA fragments containing desired mutations were digested with *Bmt*I and *Asc*I, they were ligated to pBAC-SD16 and constructs were further validated by DNA sequencing. To rescue mutated viruses, pBAC-SD16 and constructs containing mutated Nsp9 sites were introduced into MARC-145 cells using Attractene Transfection Reagent according to the manufacturer’s protocol (Qiagen, Hilden, Germany). Cell culture supernatants were harvested 3 days post-transfection and indirect immunofluorescence assay (IFA) and western blotting (WB) analyses were conducted to confirm replication of recombinant viruses. Then the cell culture supernatants serially passaged in MARC-145 cells three times; virus rescue was confirmed using whole-genome sequencing. Rescued viruses were designated rSD16-I588A, rSD16-D590A, and rSD16-L643A.

### Indirect Immunofluorescence Assay (IFA)

Virus-transfected (infected) or un-transfected (un-infected) cells were fixed with 4% paraformaldehyde at room temperature (RT) for 20 min then washed three times with PBS. Fixed cells were immediately incubated with primary antibody 6D10 diluted in PBS (pH 7.4, containing 1% BSA and 0.1% Triton X-100) at RT for 2 h. Interaction of primary antibody with corresponding target was visualized using FITC-conjugated goat anti-mouse IgG (Jackson, West Grove, PA, United States) at RT for 1 h. Cells were counterstained with 4′,6-diamidino-2-phenylindole (DAPI; Thermo Fisher Scientific) and observed via fluorescence microscopy (Zeiss, Oberkochen, Germany).

### Western Blotting

Cells were lysed using ice-cold NP-40 lysis buffer (Sorlarbio, Beijing, China) supplemented with 1 mM phenylmethylsulfonyl fluoride (PMSF) (Beyotime, Beijing, China). Cellular proteins were separated by SDS-PAGE then proteins were transferred onto polyvinylidene fluoride (PVDF) membranes (Millipore). Next, membranes were blocked by incubation in PBS buffer containing 5% skim milk supplemented with 0.1% Tween-20 then membrane-bound proteins were probed with anti-PRRSV nucleocapsid (N)-specific monoclonal antibody (Clone No. 6D10; made in-house) for 1 h at 37°C. Antibody-bound PRRSV-N protein on membranes was detected after incubation with HRP-conjugated secondary antibodies followed by development using ECL chemiluminescence substrate. Housekeeping protein glyceraldehyde-3-phosphate dehydrogenase (GAPDH) served as an internal control.

### Growth Kinetics of Mutant Viruses in MARC-145 Cells

Viral growth kinetics for wild type (WT) and mutant viruses were analyzed as previously described (Li et al., 2015). Briefly, MARC-145 cells grown in 24-well plates were infected with either WT or mutant viruses at 0.01 MOI. After a 1-h inoculation at 37°C, cell culture supernatants were replaced with fresh medium containing 3% FBS at 37°C. Culture supernatants were collected at 12, 24, 36, 48, 72, and 96 h post-infection (hpi). Viral titers of serial dilutions of harvested supernatants were calculated using the Reed-Muench method (Reed and Muench, 1938).

### Real-Time Quantitative PCR (qPCR)

MARC-145 cells infected with mutated or control viruses were harvested using TRizol Reagent (Thermo Fisher Scientific) according to the manufacturer’s instruction. Total RNA was reverse transcribed using a PrimeScript RT Kit (TaKaRa) following the manufacturer’s instructions then qPCR was performed using reactions containing SYBR Green PCR Master Mix (Roche, Mannheim, Germany) using an ABI 7500 Fast Real-Time PCR System (Thermo Fisher Scientific) with condition was: 95°C for 60 s; 40 cycles of 95°C for 15 s and 55°C for 34 s. Analysis of β-actin transcription was included in order to normalize relative expression levels of target genes as described previously (Zhang et al., 2017a,b) and calculated using the 2^–ΔΔCT^ method (Livak and Schmittgen, 2001).

### Detection of Viral Negative-Strand Genomic RNA [(−) gRNA] by Reverse Transcription PCR (RT-PCR)

MARC-145 cells were infected with mutant or wild type viruses (0.01 MOI) for 24 h prior to RNA isolation. To remove contaminating DNA, RNA preparations were further processed using a DNA-free Kit (Thermo Fisher Scientific). RT-PCR was employed to detect (−) gRNAs as previously described (Lu et al., 2011; Zhao et al., 2018). Briefly, first-strand cDNA was synthesized using forward primer F-6 (Table 1) using reverse transcriptase Superscript III (Thermo Fisher Scientific) and 2 μg of total RNA. After removing remaining RNA with RNase A (Thermo Fisher Scientific), nested PCR was performed to detect (−) gRNA using primer pairs F-6/R-683 and F-12/R-343 (Table 1) via amplification with PrimeSTAR^®^ Max DNA Polymerase (TaKaRa) with reaction program was: 30 cycles of 98°C for 30 s, 55°C for 15 s and 72°C for 10 s. Gray values for PCR products were measured using Image J software.

### Statistical Analysis

All experiments were conducted for ≥ 3 replicate samples then were independently repeated. Statistical analysis was conducted using GraphPad Prism 6, with significance assessed using Student *t*-test. A *p*-value < 0.05 was considered statistically significant.

## Results

### Preparation of Nsp9-Nb6 Complex

**FIGURE 1:**
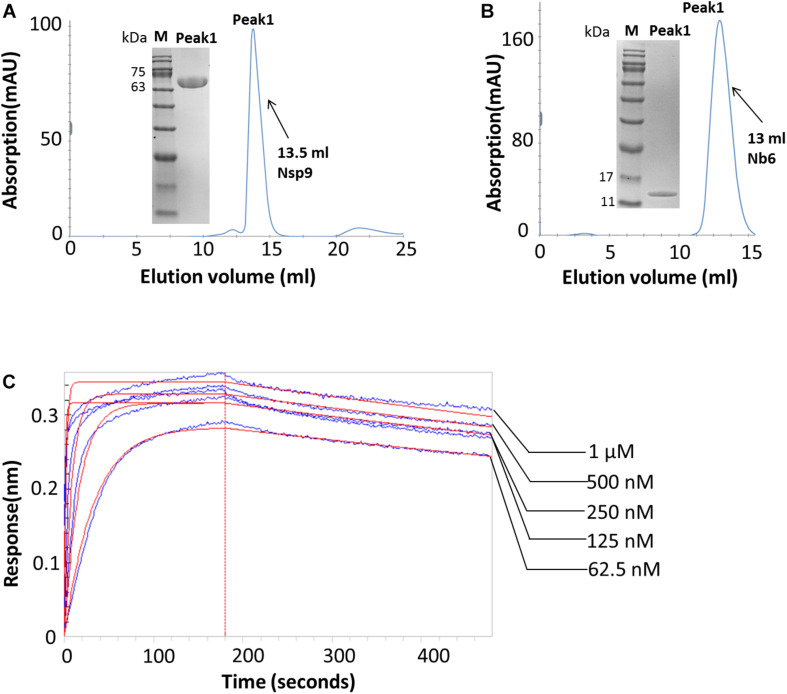
Expression and analysis of recombinant Nsp9 and Nb6. **(A)** Size-exclusion chromatography of recombinant Nsp9 on calibrated Superdex 200 increase 10/300 GL column. The target protein was eluted at a retention volume of 13.5 ml, corresponding to approximately 72 kDa as monomer. Characterization of recombinant Nsp9 after two-step purification was analyzed by SDS–PAGE (Inset), M: protein ladder (10–180 kDa, Sorlarbio, Beijing, China), Gel: 12% precast polyacrylamide gel (Item No. NP0341BOX, Thermo Fisher Scientific). **(B)** Size-exclusion chromatography of recombinant Nb6 on calibrated Superdex 75 10/300 GL column. The target protein was eluted at a retention volume of 13 ml, corresponding to approximately 15 kDa as monomer. Characterization of recombinant Nb6 after two-step purification was analyzed by SDS–PAGE (Inset), M: protein ladder (10–180 kDa, Sorlarbio, Beijing, China), Gel: 12% precast polyacrylamide gel (Item No. NP0341BOX, Thermo Fisher Scientific). **(C)** Analysis of purified Nb6 binding to Nsp9. Gradient concentrations (62.5 nM–1 μM) of Nb6 in PBS buffer (pH7.4), containing 0.02% tween-20 and 0.1% BSA were passing through the streptavidin biosensor loaded with biotinylated Nsp9, and the signals were recorded.

To discover the molecular structure of PRRSV-Nsp9, soluble Nsp9 was recombinantly expressed in a prokaryotic expression system and Nb6 was produced using a *Pichia pastoris* expression system. Both proteins were purified using metal-affinity and size-exclusion chromatography then were detected using SDS-PAGE (Figures 1A,B). Next, BLI analyses were carried out to study the interaction between recombinant Nsp9 and Nb6. As shown in Figure 1C, Nb6 bound to Nsp9 with high affinity, with a K_D_ value of approximately 10 nM. Kinetic data analysis (k_on_: 1.44 × 10^2^ M^–1^ s^–1^ and K_off_: 2.56 × 10^–5^ s^–1^) further showed that the interaction involved a quick association process and a slow dissociation process.

**FIGURE 2:**
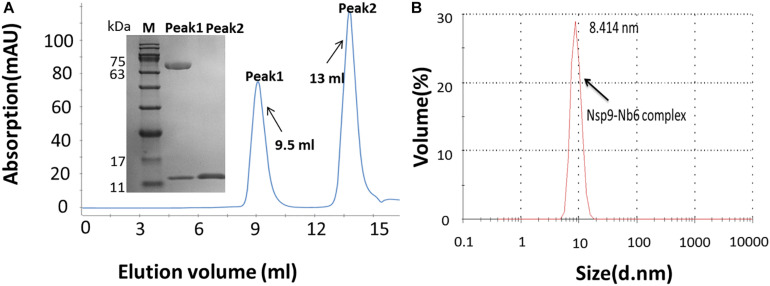
The Nsp9-Nb6 complex exists as a heterodimer. **(A)** The Nsp9-Nb6 complex was purified with high purity by calibrated Superdex 75 10/300 GL column. Two peaks were observed, with the retention volume of the Peak1 was 9.5 ml, corresponding to the heterodimer of the Nsp9-Nb6 complex, and the retention volume of the Peak 2 was about 13 ml, corresponding to the monomer of Nb6. Characterization of the complex after purification was analyzed by SDS–PAGE (Inset) M: protein ladder (10–180 kDa, Sorlarbio, Beijing, China), Gel: 12% precast polyacrylamide gel (Item No. NP0341BOX, Thermo Fisher Scientific). **(B)** DLS of Nsp9-Nb6 complex at 1.5 μM. The hydrodynamic molecular radius of the complex is 8.414 nm.

To generate the Nsp9-Nb6 complex, Nsp9 was mixed with an excess of Nb6 and the mixture was incubated at 4°C overnight. The complex was purified using gel filtration chromatography and detected via SDS–PAGE. As demonstrated in Figure 2A, two elution peaks were observed: Peak1 represented the complexed form of the two proteins and Peak2 represented unbound Nb6. Additionally, DLS analysis suggested that the complex that formed was homogeneous (Figure 2B).

### Structural Characterization of Nsp9 and Nsp9-Nb6 Complex by SAXS

**FIGURE 3:**
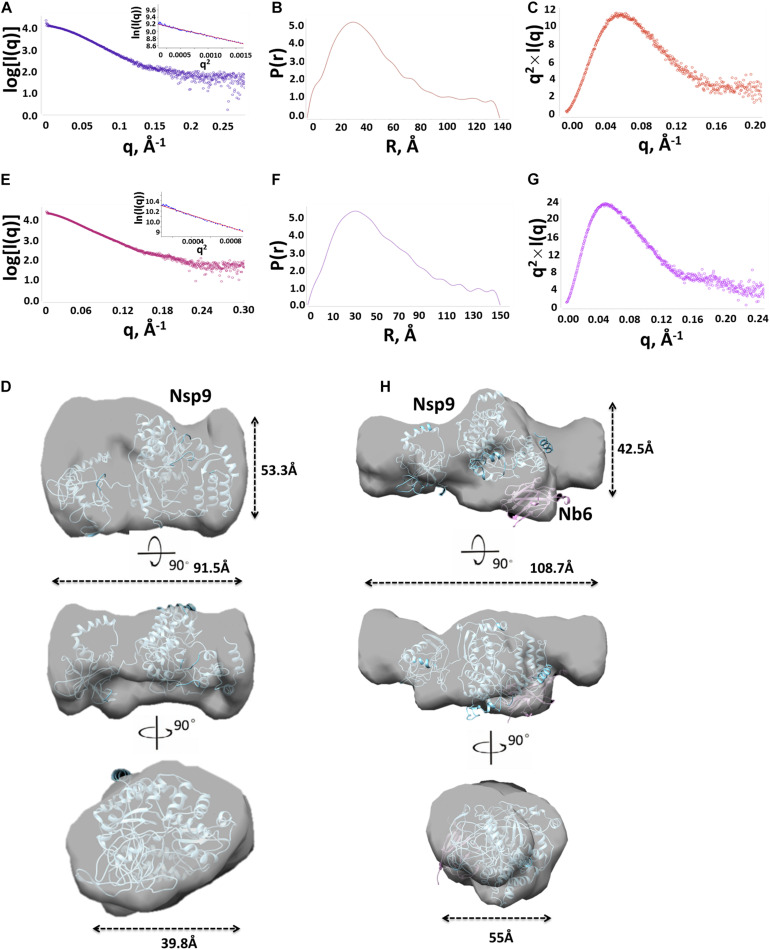
Structural characterization of Nsp9 and Nsp9-Nb6 complex by SAXS. **(A)** Experimental SAXS curve (open circles) of Nsp9 in solution. The Guinier region and the corresponding linear fitting were shown in the inset. **(B)** Pair-distance distribution function derived from the experimental curve. **(C)** Kratky plot calculated from the experimental data. Bell shaped curves indicate compact structures. **(D)** Superimposition of the experimental envelope (as shown in surface) onto the model of Nsp9 represented in cartoon. **(E)** Experimental SAXS curve (open circles) of Nsp9-Nb6 complex in solution. The Guinier region and the corresponding linear fitting were shown in the inset. **(F)** Pair-distance distribution function derived from the experimental curve. **(G)** Kratky plot calculated from the experimental data. Bell shaped curves indicate compact structures. **(H)** Superimposition of the experimental envelope (as shown in surface) onto the heterogeneous model of Nsp9 and Nb6 heterodimer represented in cartoon.

After a tremendous effort to directly generate the crystal structure of Nsp9-Nb6 complex had failed (data not shown), we next used SAXS to characterize molecular envelope structures of Nsp9 and the Nsp9-Nb6 complex. Three concentrations (2, 3 and 5 mg/ml) of Nsp9 alone were prepared and analyzed, with results showing Nsp9 protein masses of 68, 69, and 89 kDa, respectively. However, no evidence for the existence of higher order oligomers was found at any of the three protein concentrations. Considering the inherent error of the SAXS technique (Fischer et al., 2010), these results suggest that Nsp9 may exist as a monomer in solution (Table 2). Based on its highest scattering curve signal-to-noise ratio and lowest observed level of radiation damage (Figure 3A), the 2-mg/ml sample was selected for further analysis. The clear linear performance of the Guinier plot (inset image, Figure 3A) derived from this curve supported a monodispersed protein sample and yielded an Rg value of 32.4 Å, a value consistent with that obtained by scattering analysis (Rg of 33.5 Å, Table 2). The P(r) function suggested that Nsp9 was not present as a globular assembly in solution (Figure 3B), due to its maximum dimension approaching 96 Å. Kratky analysis was used to evaluate the degree of folding (Mertens and Svergun, 2010) and demonstrated (Figure 3C) a peak value around 11.5 at low q values that reverted to zero at high q values, suggesting that recombinant Nsp9 obtained here was correctly folded.

**TABLE 2:** SAXS parameters for Nsp9 and the complex of Nsp9 and Nb6.

Samples	mg/ml	Guinier		Real space			Mass, kDa	
		Rg, Å	I(0)	Rg, Å	I(0)	Dmax	SAXS, kDa	Observed, kDa
Nsp9	2	32.5	5.08e3	32.8	1.02e4	92	68	72
	3	32.4	1.02e4	33.5	1.03e4	96	69	72
	5	45.4	2.53e4	45.5	2.78e4	151	89	72
Nsp9/Nb6complex	2	41.2	1.85e4	42.8	1.88e4	122	87	90
	3	40.8	2.69e4	40.9	2.70e4	169	90	90
	5	41.4	3.13e4	43.3	3.19e4	150	96	90

To build the molecular envelope of Nsp9 from the scattering curve, it is important to know whether molecular symmetry is present in the particle, since this knowledge is essential for assessing the reliability of a molecular shape determined by SAXS. We first built the monomer molecular envelope using DAMMIF server (Franke and Svergun, 2009) by imposing a one-fold symmetry constraint onto Nsp9 then the DAMAVER program was used to generate an average molecular envelope. The model of Nsp9 was predicted using the I-TASSER server and built onto the averaged molecular envelope obtained using CHIMERA (Pettersen et al., 2004). Using this method, the molecular envelope was determined to be asymmetric, with a barrel shape of overall dimensions of approximately 91.5 Å × 53.3 Å × 39.8 Å (Figure 3D).

Meanwhile, the molecular envelope of the Nsp9-Nb6 complex was built from the scattering curve in parallel. First, SAXS results of the complexes at three concentrations yielded molecular masses of 87, 90, and 96 kDa, respectively, indicating that the Nsp9-Nb6 complex was a heterodimer in solution. Next, the 5 mg/ml sample was chosen for further analysis, due to its higher scattering curve signal-to-noise ratio and absence of radiation damage; the resulting Guinier plot (Figure 3E) exhibited a clearly linear presentation that indicated complexed protein was present in a monodispersed state (Figure 3E). The Rg value was 41.4 Å which was consistent with the value obtained using Real Space analysis (Rg of 43.3 Å, Table 2). The P(r) function suggested that the Nsp9-Nb6 complex was not present as a globular assembly in solution, due to its maximum dimension approaching 150 Å (Figure 3F). Kratky analysis give a peak value of about 24 at low q values and zero at high q values, suggesting that the complex was correctly folded (Figure 3G). The average molecular envelope was obtained using DAMMIF server results based on a one-fold symmetry constraint followed by analysis using the DAMAVER program. Meanwhile, the Nsp9-Nb6 complex structure was predicted using the online I-TASSER server then the predicted structural models were built onto the averaged molecular envelope using CHIMERA. Ultimately, the molecular envelope of the complex was also shown to be asymmetric, with a Z-shape with overall dimensions of approximately 108.7 Å × 55 Å × 42.5 Å (Figure 3H).

**FIGURE 4:**
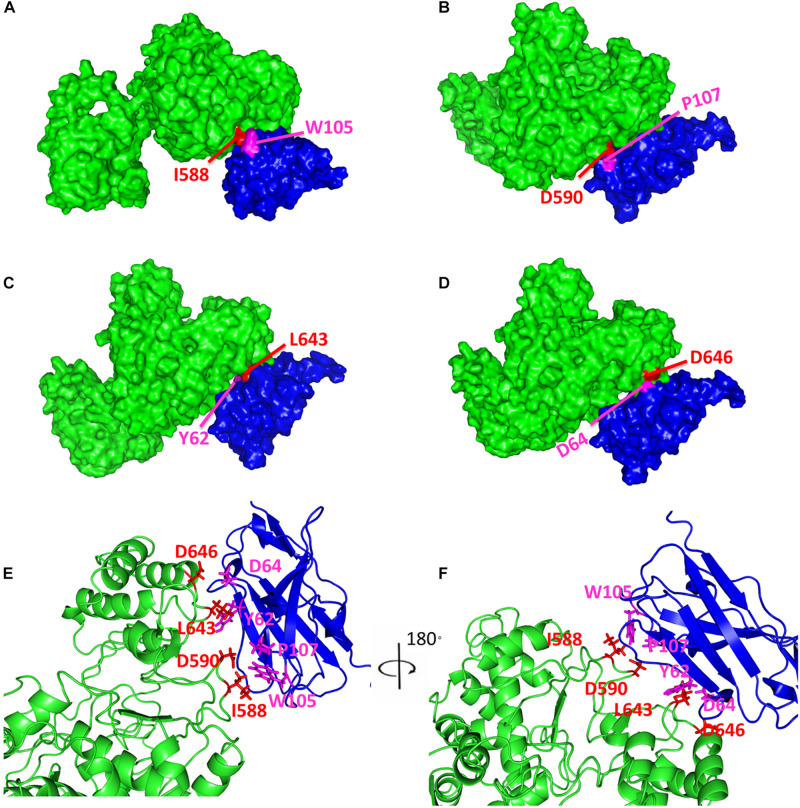
Predicted model of Nsp9-Nb6 complex. The surface representations of Nsp9-Nb6 complex are shown in **(A–D)**. Nsp9 is colored in green, Nb6 is in blue, Ile588 **(A)**, Asp590 **(B)**, Leu643 **(C)** and Asp646 **(D)** in Nsp9 are highlighted in red, Trp105 **(A)**, Pro107 **(B)**, Tyr62 **(C)** and Asp64 **(D)** in Nb6 are highlighted in magentas. **(E,F)** The predicted complex of Nsp9 and Nb6 are shown in cartoon diagrams.

As demonstrated above, it was notable that a molecular envelope shape change occurred when Nb6 bound to Nsp9, with the shape becoming longer and a protrusion forming in the middle of the shape that were consistent with Nb6 represented as the protrusion of the complex. Ultimately, the final secondary structure models fitted tightly together with the SAXS molecular envelope, with complex formation shown to involve Nsp9 aa residues Ile588, Asp590, Leu643 and Asp646 and Nb6 aa residues Trp105, Pro107, Tyr62 and Asp64 (Figure 4).

### Screening for Amino Acid Residues Involved in the Binding Interaction Between Nsp9 and Nb6

**FIGURE 5:**
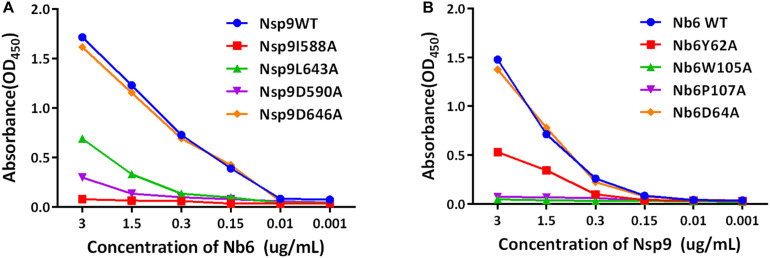
Identification of residues involved in the interaction between Nsp9 and Nb6 by ELISAs. **(A)** Binding efficiency of the WT and mutant Nsp9 to Nb6. 96-well microtiter plates were coated with the WT and mutant Nsp9 (200 ng/well). After incubation with biotinylated Nb6, bound proteins were detected by HRP-conjugated streptavidin. **(B)** Binding efficiency of the WT and mutant Nb6 to Nsp9. 96-well microtiter plates were coated with the WT and mutant Nb6 (200 ng/well). After incubation with biotinylated Nsp9, bound proteins were detected by HRP-conjugated streptavidin. The binding efficiency was calculated on the basis of optical density (OD_450_). Assays were performed in triplicate and data are presented as means ± SD.

After the abovementioned structure model was used to predict putative key Nsp9 residues (aa 588, 590, 643, 646) required for interacting with Nb6, site-directed mutagenesis was carried out to generate a panel of recombinant single-site Nsp9 mutants with aa substitutions of those key residues. Next, mutant Nsp9 proteins were expressed, isolated, and then tested for binding to biotinylated Nb6 via ELISA. Based on ELISA results, the interaction of mutant protein Nsp9-D646A with Nb6 was similar to that of WT Nsp9 with Nb6. However, the Nsp9 I588A mutant exhibited a complete loss of binding to Nb6, while D590A and L643A mutants exhibited only impaired binding to Nb6 (Figure 5A). Conversely, single-site Nb6 mutants were also studied. Although the binding of Nb6-D64A was similar to the WT control, Nb6-W105A and Nb6-P107A mutants did not bind to Nsp9 at all, while Nb6-Y62A showed partial binding (Figure 5B). When considered together, these results suggest that Nsp9 aa residues 588, 590, and 643 interacted with Nb6 aa residues 105, 107, and 62 in a reciprocal fashion to form the Nsp9-Nb6 complex.

### Mutations of Residues 588 and 643 of PRRSV-Nsp9 Affect Viral Replication *in vitro*

**FIGURE 6:**
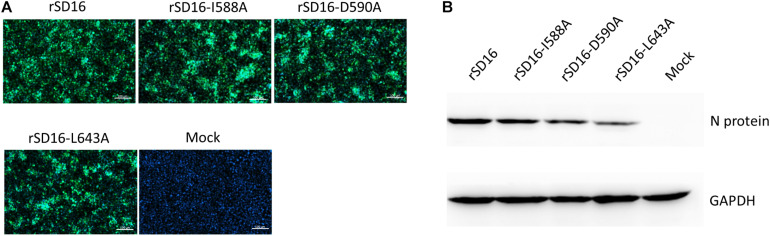
Rescue of recombinant PRRSV bearing Nsp9 mutation. **(A)** MARC-145 cells transfected with DNA-launched infectious clones of wild type PRRSV-SD16 strain (rSD16) or infectious clones bearing indicated mutations in the putative interaction sites (I588A, D590A, L643A) between Nsp9 and Nb6 on rSD16 backbone. IFA were performed to detect PRRSV-N protein expression and visualized by FITC-conjugated goat anti-mouse IgG. Overlay images were visualized for cell nuclei (blue) and N protein (green) with mock-transfected cells were included as control. The scale bars are 100 μm. **(B)** WB analysis of PRRSV-N protein expression in MARC-145 cells transfected with DNA-launched infectious clones of indicated mutants or wild type virus. Mock-transfected cells were used as control.

To characterize the involvement and biological significance of the abovementioned residues in PRRSV replication, a reverse genetics-based system was employed using an infectious clone of HP-PRRSV strain SD16 (pBAC-SD16) as the backbone for mutagenesis. However, only reverse-genetically engineered virus with Nsp9 substitutions I588A, D590A or L643A could be rescued, with replication of recombinant viruses confirmed based on PRRSV-N protein expression in MARC-145 cells using IFA and WB (Figure 6).

**FIGURE 7:**
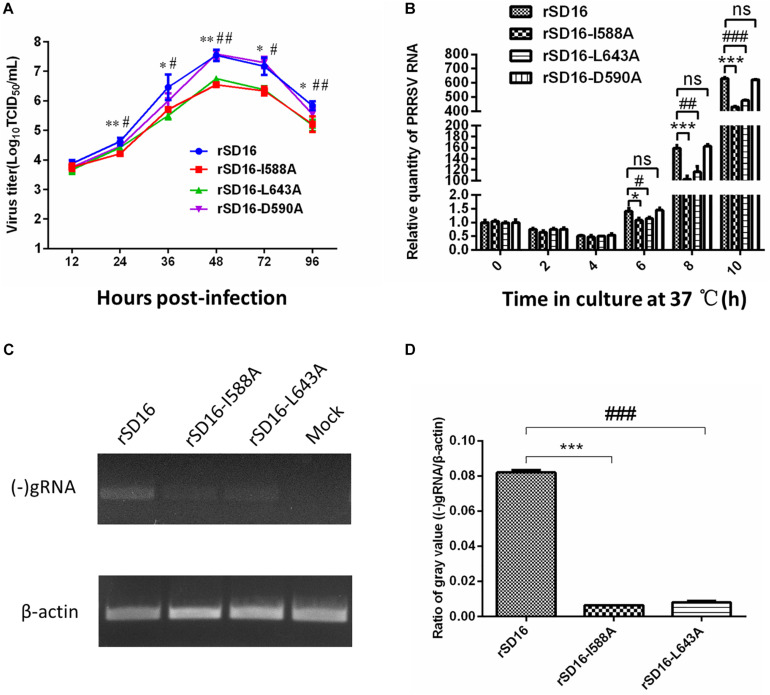
The effect of Nsp9 mutants on PRRSV replication. **(A)** The growth kinetics of the rescued recombinant viruses bearing different mutations in Nsp9 were analyzed. **(B)** Relative levels of PRRSV RNA of recombinant viruses were further analyzed for early time points. Data represent means ± SD from three independent experiments. Asterisk (*) indicates a significant difference between rSD16 and rSD16-I588A (*p < 0.05; **p < 0.01; ***p < 0.001). Pound (^#^) shows a significant difference between rSD16 and rSD16-L643A (^#^*p* < 0.05; ^##^*p* < 0.01; ^###^*p* < 0.001); ns, not significant. **(C)** Total cellular RNAs were extracted from recombinant virus-transfected MARC-145 cells at 24 hpi, and then subjected to reverse transcribed-PCR (RT-PCR) and agarose gel electrophoresis. The mRNA level of β-actin was included as reference for total RNA input. **(D)** The ratio of the gray value for PCR products of (−) gRNA/β-actin calculated using Image J software. Asterisk (*) indicates a significant difference between rSD16 and rSD16-I588A (***p < 0.001). Pound (^#^) shows a significant difference between rSD16 and rSD16-L643A (^###^*p* < 0.001).

**FIGURE 8:**
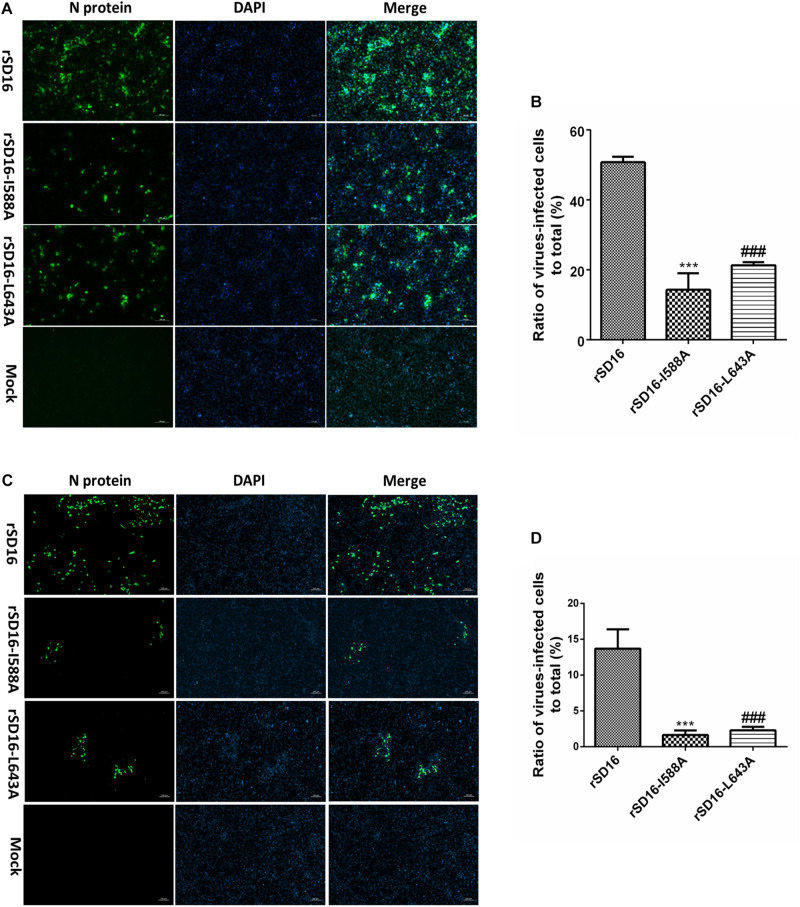
Recombinant PRRSV viruses bearing mutations of residues 588 and 643 of Nsp9 impaired PRRSV replication *in vitro*. MARC-145 Cells **(A)** and CRL-2843-CD163 cells **(C)** were infected with mutated viruses or wild type rSD16 (0.1 MOI). IFA was performed at 48 hpi with the monoclonal antibody recognized PRRSV-N protein (Clone No. 6D10) and visualized by FITC-conjugated goat anti-mouse IgG. Overlay images were visualized for cell nuclei (blue) and N protein (green). The scale bars are 100 μm. **(B,D)** The ratio of virus-infected cells/total cells calculated using Image J software. Asterisk (*) indicates a significant difference between rSD16 and rSD16-I588A (***p < 0.001). Pound (^#^) shows a significant difference between rSD16 and rSD16-L643A (###*p* < 0.001).

To compare these mutants to wild type virus, mutant virus growth kinetics were characterized in MARC-145 cells. As shown in Figure 7A, titers of all viruses reached peak levels at 48 hpi then decreased from 48 hpi to 96 hpi, with mutant virus rSD16-D590A and rSD16 exhibiting similar growth kinetics. At 24 hpi, average rSD16-I588A and rSD16-L643A virus titers reached 4.208 log_10_TCID_50_/ml and 4.417 log_10_TCID_50_/ml, respectively, that were both significantly lower than the rSD16 titer. At 36 hpi, mutant viruses rSD16-I588A and rSD16-L643A titers were 5.71 log_10_TCID_50_/ml and 5.5 log_10_TCID_50_/ml, respectively, which were also markedly lower than the rSD16 titer. Virus titers of mutants rSD16-I588A and rSD16-L643A, measured at 48 h, 72 h and 96, attained similar levels at 24 h and 36 h as compared to wild type virus or rSD16-D590A (Figure 7A). Meanwhile, detailed analysis of viral RNA copy numbers at early time points after MARC-145 cell infected with mutant viruses and wild type virus demonstrated similar trends as well (Figure 7B), implying that mutation of Nsp9 residues at positions 588 and 643 (I588A and L643A) could potentially impair PRRSV RNA replication. Therefore, to further investigate viral genome replication, intracellular RNAs were isolated from transfected MARC-145 cells and analyzed via RT-PCR analysis for (−) gRNA. Based on the semi-quantification method, rSD16-I588A and rSD16-L643A (−) gRNA synthesis levels were approximately 12-fold and 10-fold lower than the wild type rSD-16 level, respectively (Figures 7C,D), thus supporting our speculation that I588A and L643A Nsp9 mutations may reduce viral RdRp activity. Meanwhile, further evidence was obtained using fresh MARC-145 cells and CRL-2843-CD163 cells inoculated with recombinant viruses (at a MOI of 0.1) via monitoring of viral replication by immunofluorescence after incubating inoculated cells with monoclonal antibody specific for PRRSV-N protein (Figures 8A,C). A quick enumeration of immunofluorescent cell numbers revealed reduced replication levels of recombinant viruses bearing I588A or L643A mutations relative to wild type virus levels after inoculation of cells with the same initial dose of virus (Figures 8B,D). Taken together, the abovementioned results indicate that the mutations of Nsp9 residues 588 and 643 decreased replication of SD16 strain PRRSV *in vitro.*

### Amino Acid Residue 588 of Nsp9 Is Conserved in Both PRRSV Genotypes

**FIGURE 9:**
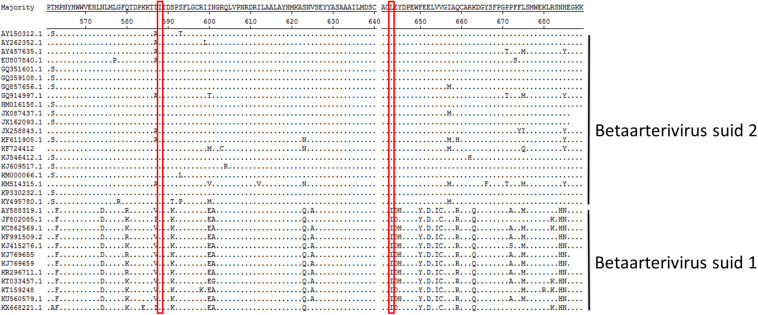
The sequence alignment of aa residues 561-689 in PRRSV Nsp9. The sequences of Nsp9 from the 32 PRRSV strains were pulled from Genbank and then analyzed using Lasergene. The residues at 588 and 643 of Nsp9 are shown in red frame with dots indicating identical amino acid residues.

Due to the fact that our data suggested that Nsp9 amino acid resides 588 and 643 were crucial for PRRSV replication in cells, Nsp9 amino acid sequences for the aa561-689 region from 32 PRRSV strains (12 *Betaarterivirus suid 1* isolates and 20 *Betaarterivirus suid 2* isolates) were obtained from GenBank and aligned using the Clustal W module of Lasergene 7.1 (DNASTAR, Inc.). The results showed that residue Ile at position 588 was conserved among strains across both PRRSV genotypes, while the Leu residue at 643 was only conserved in the 20 *Betaarterivirus suid 2* sequences analyzed here, but not among *Betaarterivirus suid 1* isolates analyzed (Figure 9). Although our reverse genetics-based system was based on the *Betaarterivirus suid 2* SD-16 strain, our data suggest that these two highly conserved Nsp9 residues (588 and 643) may be crucial for PRRSV replication and thus may serve as novel targets of anti-PRRSV therapies to achieve viral attenuation *in vivo*.

## Discussion

Since its discovery, PRRS has caused significant economic losses to the global swine industry due to the ineffectiveness of vaccinations and the lack of efficient antiviral strategies. In China this situation has worsened since 2006, when HP-PRRSV emerged there. Although tremendous efforts have been dedicated to elucidating PRRSV pathogenesis, contributing factors underlying viral virulence remain elusive, including regulatory mechanisms involving host immunity, viral neutralizing targets and protective immune responses against PRRSV. In early reports, antagonism of host innate immunity was considered the major underlying factor for PRRSV virulence, as several PRRSV proteins, such as Nsp1α, Nsp1β, Nsp2, Nsp4, Nsp11 and PRRSV-N, were demonstrated to block IFNs induction and IFN-activated JAK/STAT signaling (Chen et al., 2010; Li et al., 2010; Patel et al., 2010; Sun et al., 2010; Wang et al., 2013a,b; Wang and Zhang, 2014; Yang et al., 2017) or the NF-κB signaling pathway (Huang et al., 2014). However, this explanation for viral virulence has been challenged by discoveries of PRRSV isolates that maintain a virulent phenotype *in vivo* but which also induce robust IFNs production and other innate immune response *in vitro* (Nan et al., 2012; Sun et al., 2016). Therefore, it appears that PRRSV proteins other than innate immunity antagonists also may be involved in PRRSV virulence and pathogenesis.

Since the emergence of HP-PRRSV in China, researchers have conducted several studies to determine viral factors responsible for increased virulence using reverse genetics-based approaches. Unexpectedly, swapping of genetic fragments between infectious clones of highly pathogenic and low pathogenic clones derived from HP-PRRSV isolates has indicated that Nsp9 and Nsp10 are contributing factors to HP-PRRSV virulence *in vivo* (Li et al., 2014). Moreover, a more recent report has demonstrated that two Nsp9 aa residues (aa519 and aa544) contribute to pathogenicity of HP-PRRSV *in vivo* by improving PRRSV replication efficiency (Zhao et al., 2018). Therefore, additional investigations of PRRSV-Nsp9 are required to enhance our understanding of PRRSV pathogenesis.

As a PRRSV-specific RdRp, Nsp9 is crucial for viral RNA replication and transcription (Beerens et al., 2007). It has been demonstrated that both viral and cellular factors associate with Nsp9 to regulate viral RNA synthesis, including PRRSV-N protein and host DHX9 RNA helicase and retinoblastoma protein (Dong et al., 2014; Liu et al., 2016). However, the crystal structure of Nsp9 remains unsolved; even our own persistent efforts have failed to produce a high resolution crystal structure of PRRSV-Nsp9. Therefore, we propose that a protein-interacting partner may be needed to stabilize Nsp9 in solution and act as a chaperone to promote suitable crystal formation. In our previous work, we developed the PRRSV-Nsp9-specific nanobody Nb6 to serve as such a chaperone. Nanobodies, unlike conventional antibodies, contain a long stable convex loop within the complementarity determining region 3 (CDR3) that permits them to penetrate deeply into interior pockets of antigens, such as active sites of enzymes (Hassanzadeh-Ghassabeh et al., 2013). Notably, Nbs can also stabilize proteins in certain conformations, with past reports demonstrating successful use of Nbs for inducing lattice formation during crystallization (Lam et al., 2009; Rasmussen et al., 2011; Park et al., 2012). Therefore, recombinant Nsp9 and corresponding Nb6 were produced here to generate the Nsp9-Nb6 complex. However, although crystallization of the Nsp9-Nb6 complex was demonstrated, we ultimately could not obtain suitable crystals for crystallographic analysis. Consequently, we used SAXS to determine the molecular envelope of Nsp9 and the Nsp9-Nb6 complex. SAXS is a well-established technique for generating low-resolution structures of macromolecules in solution (Svergun, 2010), especially for characterization of solution-based protein-to-protein or protein-to-nucleic acid complexes (Gabel, 2015).

Our SAXS data indicate that Nsp9 was monomeric in solution, with the monomer assuming an asymmetric barrel shape (Figure 3D). For Nsp9-Nb6 heterodimers, the molecular envelope was asymmetric and assumed a Z-shape (Figure 3H) and was not present as a globular assembly or fully folded protein in solution. Notably, a conformational change of the molecular envelope occurred when Nb6 bound to Nsp9 that generated a conformation that differed from that of Nsp9 alone. In the Nsp9-Nb6 complex, the molecular envelope was longer and a protrusion was present in the middle of the shape (Figures 3D,H). Therefore, we speculated that Nb6 was situated at the location of the protrusion. In our model, complex formation involved residues Ile588, Asp590, Leu643 and Asp646 of Nsp9 and Trp105, Pro107, Tyr62 and Asp64 of Nb6 (Figure 4). Next, we carried out site-directed mutagenesis of putative amino acid residues responsible for the Nsp9-Nb6 interaction then conducted ELISAs to detect interactions between Nsp9 mutants and Nb6 and interactions between Nb6 mutants and Nsp9. Our result demonstrated that residues at positions 588, 590, 643 of Nsp9 and at positions 62, 105, 107 of Nb6 were involved in the Nsp9-Nb6 interaction (Figure 5). These results are consistent with our previous results demonstrating that Nb6 interacting regions spanned two discontinuous regions located within the C-terminal region of Nsp9 (residues 454-551 and 599-646) (Wang et al., 2019).

Importantly, our previous study had demonstrated that Nb6 blocked PRRSV replication by targeting Nsp9 both in MARC-145 cells and porcine alveolar macrophages (PAMs). In the present study, we identified critical amino acid residues involved in the Nsp9-Nb6 interaction and hypothesized that Nsp9 residues 588, 590 and 643 may affect viral replication. To determine the importance of these residues to PRRSV function, reverse genetics-based mutagenesis was applied to generate mutated viruses bearing a single-mutation or double-mutations. Ultimately, we observed that double-mutations of Nsp9 prevented PRRSV replication rescue of recombinant virus *in vitro*, implying that these residues are crucial for PRRSV functions (data not shown). Meanwhile, viral replication levels and (−) gRNA synthesis levels for single-mutant viruses rSD16-I588A and rSD16-L643A were lower than corresponding wild type rSD16 levels, indicating that mutations altering Nsp9 amino acids 588 or 643 reduced PRRSV RNA replication.

Based on structural models, PRRSV Nsp9 contains an RdRp domain within its C-terminal sequence (Gorbalenya et al., 1989). Although both residues required for Nb6-Nsp9 interaction (Ile588 and Leu643) are located within the Nsp9 C terminus, their locations are far from the enzymatic center of the RdRp domain, implying that the interaction of Nb6 with Nsp9 may alter the 3-dimensional structure of Nsp9 to block RdRp activity. Alternatively, it is also possible that the interaction between Nb6 and Nsp9 interferes with associations of Nsp9 with other viral or cellular proteins required for viral RTC assembly, although further investigations are required to test these speculations. Nevertheless, sequence alignments generated here indicate that residue Ile588 of PRRSV-Nsp9 is conserved among all *Betaarterivirus suid 1* and *Betaarterivirus suid 2* isolates analyzed, while residue Leu643 is only conserved in *Betaarterivirus suid 2* isolates. These observations suggest that these residues are crucial for PRRSV replication across and within PRRSV genotypes, respectively. As many viruses enhance their pathogenicity by increasing viral replication efficiency (Hanley et al., 2001; Watanabe et al., 2009), it would be interesting to know whether mutation of these residues would attenuate phenotypic PRRSV virulence *in vivo*. Indeed, a previous report has suggested that mutation of certain Nsp9 residues leads to an attenuated phenotype (Xu et al., 2018; Zhao et al., 2018). However, more evidence is needed than that which has been collected so far (including results of this work) to enhance our understanding of PRRSV Nsp9.

In conclusion, the application of SAXS to understand molecular envelopes of Nsp9 and the Nsp9-Nb6 complex has permitted us to identify two novel residues of Nsp9 required for the Nsp9-Nb6 interaction. Moreover, results obtained here have further elucidated the molecular mechanism whereby Nb6 acts as a novel PRRSV inhibitor while also revealing crucial residues conserved across PRRSV genotypes or within *Betaarterivirus suid 2* that are essential for PRRSV replication *in vitro*. These findings enhance our understanding of PRRSV replication and will likely facilitate development of anti-PRRSV drugs and future vaccines.

## Data Availability Statement

The original contributions presented in the study are included in the article/supplementary material, further inquiries can be directed to the corresponding author/s.

## Author Contributions

YW and RL performed the experiment, analyzed the data, and drafted the manuscript. SQ, JW, HL, ZL, and HM contributed to protein expression. LY, HR, and MW contributed to PRRSV replication assay. JH and JS contributed to the immunofluorescence assay. YN, GZ, and E-MZ conceived the study, carried out additional analyses, and finalized the manuscript. All authors contributed to the revising manuscript.

## Conflict of Interest

The authors declare that the research was conducted in the absence of any commercial or financial relationships that could be construed as a potential conflict of interest.

## Footnotes

1 http://zhanglab.ccmb.med.umich.edu/I-TASSER/
